# Granulocyte colony-stimulating factor treatment ameliorates lupus nephritis through the expansion of regulatory T cells

**DOI:** 10.1186/s12882-016-0380-x

**Published:** 2016-11-15

**Authors:** Ji-Jing Yan, Enkthuya Jambaldorj, Jae-Ghi Lee, Joon Young Jang, Jung Min Shim, Miyeun Han, Tai Yeon Koo, Curie Ahn, Jaeseok Yang

**Affiliations:** 1 Transplantation Research Institute, Seoul National University College of Medicine, Seoul, 03080 Republic of Korea; 2 Department of Internal Medicine, Seoul National University College of Medicine, Seoul, 03080 Republic of Korea; 3 Transplantation Center, Seoul National University Hospital, Seoul, 03080 Republic of Korea; 4 Department of Surgery, Seoul National University Hospital, Seoul, 03080 Republic of Korea

**Keywords:** Granulocyte colony-stimulating factor, Lupus nephritis, Regulatory T cells

## Abstract

**Background:**

Granulocyte colony-stimulating factor (G-CSF) can induce regulatory T cells (Tregs) as well as myeloid-derived suppressor cells (MDSCs). Despite the immune modulatory effects of G-CSF, results of G-CSF treatment in systemic lupus erythematosus are still controversial. We therefore investigated whether G-CSF can ameliorate lupus nephritis and studied the underlying mechanisms.

**Methods:**

NZB/W F1 female mice were treated with G-CSF or phosphate-buffered saline for 5 consecutive days every week from 24 weeks of age, and were analyzed at 36 weeks of age.

**Results:**

G-CSF treatment decreased proteinuria and serum anti-dsDNA, increased serum complement component 3 (C3), and attenuated renal tissue injury including deposition of IgG and C3. G-CSF treatment also decreased serum levels of BUN and creatinine, and ultimately decreased mortality of NZB/W F1 mice. G-CSF treatment induced expansion of CD4^+^CD25^+^Foxp3^+^ Tregs, with decreased renal infiltration of T cells, B cells, inflammatory granulocytes and monocytes in both kidneys and spleen. G-CSF treatment also decreased expression levels of MCP-1, IL-6, IL-2, and IL-10 in renal tissues as well as serum levels of MCP-1, IL-6, TNF-α, IL-10, and IL-17. When Tregs were depleted by PC61 treatment, G-CSF-mediated protective effects on lupus nephritis were abrogated.

**Conclusions:**

G-CSF treatment ameliorated lupus nephritis through the preferential expansion of CD4^+^CD25^+^Foxp3^+^ Tregs. Therefore, G-CSF has a therapeutic potential for lupus nephritis.

**Electronic supplementary material:**

The online version of this article (doi:10.1186/s12882-016-0380-x) contains supplementary material, which is available to authorized users.

## Background

Systemic lupus erythematosus (SLE) is an autoimmune disease in which altered T cell function and polyclonal B cell activation followed by autoantibody production including anti-double stranded DNA antibodies (anti-dsDNA), thus, leading to immune complex deposition in multiple organs, particularly in the kidney. These depositions drive local inflammatory responses that can lead to tissue damage and clinical disease [1, 2].

Granulocyte colony-stimulating factor (G-CSF) is a growth factor for neutrophils, that can accelerate neutrophil reconstitution after bone marrow suppression and activate effector functions of mature neutrophils [3]. G-CSF also stimulates proliferation, differentiation, and peripheral mobilization of hematopoietic stem cells [3]. Furthermore, G-CSF has been reported to modulate both T cell and innate immune responses. G-CSF can induce Tr1 cells in vitro, and mobilize CD4^+^CD25^+^Foxp3 regulatory T cells (Tregs) [4, 5]. In addition, G-CSF can mobilize human tolerogenic dendritic cells and induce human semi-mature dendritic cells, and thereby induce type 1 regulatory T (Tr1) cells through interleukin (IL)-10 release [6, 7]. G-CSF suppresses production of IL-1β, IL-12, interferon (IFN)-γ, and tumor necrosis factor (TNF)-α, but increases serum levels of IFN-α and IL-10 [7, 8]. Recently, G-CSF was also reported to induce CD11b^+^Gr-1^+^ myeloid-derived suppressor cells (MDSC), a subset of innate-like suppressor cells [9, 10]. MDSCs are a heterogeneous population of immature myeloid cells that can suppress both T cells and natural killer (NK) cells and thereby suppress autoimmunity as well as tumor immunity [10, 11]. MDSCs are divided into two subsets, granulocytic MDSCs (CD11b^+^Ly6G^+^Ly6C^low^), and monocytic MDCSs (CD11b^+^Ly6G^-^Ly6C^high^) [10]. However, CD11b^+^Ly6G^+^Ly6C^low^ and CD11b^+^Ly6G^-^Ly6C^high^ cells could be inflammatory granulocytes and inflammatory monocytes, respectively, because these populations share surface markers with MDSCs.

Despite the immunomodulatory effects of G-CSF, results of G-CSF treatment in SLE are still controversial. Low-dose G-CSF treatment accelerated lupus nephritis in MRL lymphoproliferation (MRL/lpr) strain mice and increased autoantibody production in B6.S*le1.Sle2.Sle3* spontaneous mouse model of lupus [12, 13]. In contrast, high-dose G-CSF treatment prevented lupus nephritis and delayed mortality [13]. In patients, G-CSF induced disease flares in both lupus nephritis and cutaneous lupus [14, 15]. These controversial results require further study to confirm the role of G-CSF in lupus nephritis and to determine the involved mechanisms. In this study, we investigated whether G-CSF can ameliorate lupus nephritis in a NZB/W F1 mouse lupus model, and examined the related mechanisms.

## Methods

### Animals and treatment regimens

NZB/W F1 mice spontaneously develop a disease closely resembling human SLE [16]. Female NZB/W F1 mice were purchased from SLC Inc. (Hamamatsu, Japan) and housed under the pathogen-free conditions. The experimental group was injected subcutaneously with recombinant human G-CSF (Grasin, Kyowa Kirin, Korea) for 5 consecutive days every week from 24 weeks of age, at a dose of 250 μg/kg/day during 12 weeks. In low-dose experiments, 250 μg/kg/day of human G-CSF was administered 3 times a week for 12 weeks from 24 weeks of age. The control group received only phosphate-buffered saline (PBS) injection. For Treg depletion, depleting anti-CD25 antibodies (PC61, Bio X Cell, West Lebanon, NH, USA) were administered at a dose of 0.5 mg 3 times a week from 33 to 36 weeks.

### Measurement of proteinuria, renal function, anti-dsDNA, complement component 3 (C3), and cytokines

Spot urine proteinuria was measured using protein reagent strips (URiSCAN; Yongdong Pharmaceutical Co., Seoul, Korea) once per week for 12 weeks. The measurement was semi-quantitative: 0 = none or trace amount of proteinuria, 1+, 30–100 mg/dL; 2+, 100–300 mg/dL; 3+, 300–1000 mg/dL; 4+, ≥ 1000 mg/dL. Urine albumin concentrations were measured using a mouse albumin ELISA kit (Alpco Diagnostics, Salem, NH, USA) and normalized to urine creatinine concentrations. Serum levels of BUN and creatinine were measured at 32 and 36 weeks using QuantiChrom urea and creatinine assay kits (BioAssay Systems, Hayward, CA, USA) [17]. Serum concentration of mouse anti-dsDNA and C3 were measured using ELISA kits (Alpha Diagnostic International, San Antonio, TX, USA; Abcam, Cambridge, MA, USA) at 36 weeks. Levels of monocyte chemoattractant protein-1 (MCP-1) and cytokines (IL-6, TNF-α, IL-2, IFN-γ, IL-10, IL-4 and IL-17) were measured at 36 weeks in both serum and renal tissues using cytometric bead array kits (BD Biosciences, San Diego, CA, USA).

### Renal histologic analysis

Paraffin sections of fixed kidneys were stained with Periodic Acid-Schiff’s (PAS) stain kit, and evaluated according to described protocol [18, 19]. Briefly, glomerular pathology was evaluated by assessing 20 glomerular cross-sections (gcs) per kidney, and each glomerulus was scored on a semiquantitative scale: 0 = normal (35–40 cells/glomerular cross-sections, gcs); 1 = mild (glomeruli with few lesions, slight proliferative changes, mild hypercellularity, 41–50 cells/gcs); 2 = moderate (glomeruli with moderate hypercellularity, 50–60 cells/gcs, segmental and/or diffuse proliferative changes, hyalinosis); 3 = severe (glomeruli with segmental or global sclerosis, and/or exhibiting severe hypercellularity (>60 cells/gcs), necrosis, crescent formation). Interstitial/tubular pathology was assessed semiquantitatively on a scale of 0–3 in 10 randomly selected high-power fields. We determined the largest and average number of infiltrates and damaged tubules and subsequently adjusted the grading system accordingly: 0, normal; 1, mild; 2, moderate; 3, severe. Perivascular cellular accumulation was determined semiquantitatively by scoring the number of cell layers surrounding the majority of vessel walls (0, none; 1, < 5; 2, 5–10; 3, ≥ 10 cell layers).

Kidney cryostat sections were stained with goat anti-mouse IgG (Sigma-Aldrich) or rabbit anti-mouse C3 (Abcam) for 4 h. Then, they were incubated at room temperature with Alexa Fluor 488 donkey anti-goat IgG or Alexa Fluor 568 donkey anti-rabbit IgG (Molecular Probe; Invitrogen USA) for 1 h. Deposition of IgG and C3 within the peripheral glomerular capillary walls and the mesangium was measured as the mean fluorescence in 10 glomeruli per mouse. Scores were assigned based on the intensity of IgG/C3 deposition (0–3+), where 0 represents no deposition and 3 denotes intense deposition [20]. All histologic analysis was performed by two independent pathologists blinded to the treatment group.

### Flow cytometry

Renal leukocytes and splenocytes were pretreated with anti-mouse CD16/32 (clone 2.4G2) to block nonspecific Fc binding. Kidney leukocytes were identified by labeling cells with CD45 (30-F11). The fluorochrome-conjugated antibodies (BD Biosciences or eBioscience) were used for flow cytometric analysis: CD45 (30-F11), CD3 (145-2C11), CD4 (RM4-5), CD8 (53-6.7), CD25 (ebioPC61), CD44 (IM7), CD69 (H1.2 F3), Foxp3 (ebio), CD11b (M1/70), Gr-1 (RB6-BC5, ebio), Ly6G (RB6-8c5), Ly6C (AL-21), CD19 (eBio1D3), CD138 (281-2), CXCR5 (SPRCL5), PD-1 (J43), NK1.1 (PK136). Appropriate fluorochrome-conjugated isotype-matched irrelevant monoclonal antibodies were used as negative controls. 7-aminoactinomycin D was also added to distinguish between live and dead cells. The stained cells were analyzed using BD FACSCanto II (BD Biosciences).

### Statistical analysis

Data were expressed as the mean ± standard error of the mean (SEM). Student’s *t*-test was used to compare means. Survival rates were analyzed using the Kaplan-Meier method and compared by the log-rank test. Difference in proteinuria during treatment was analyzed using generalized estimating equations. *P* values of less than 0.05 were considered statistically significant. All data were analyzed using SPSS v.22.0 software (SPSS Inc., Chicago, IL, USA).

## Results

### G-CSF treatment ameliorated lupus-like disease in NZB/W F1 mice

**Fig. 1:**
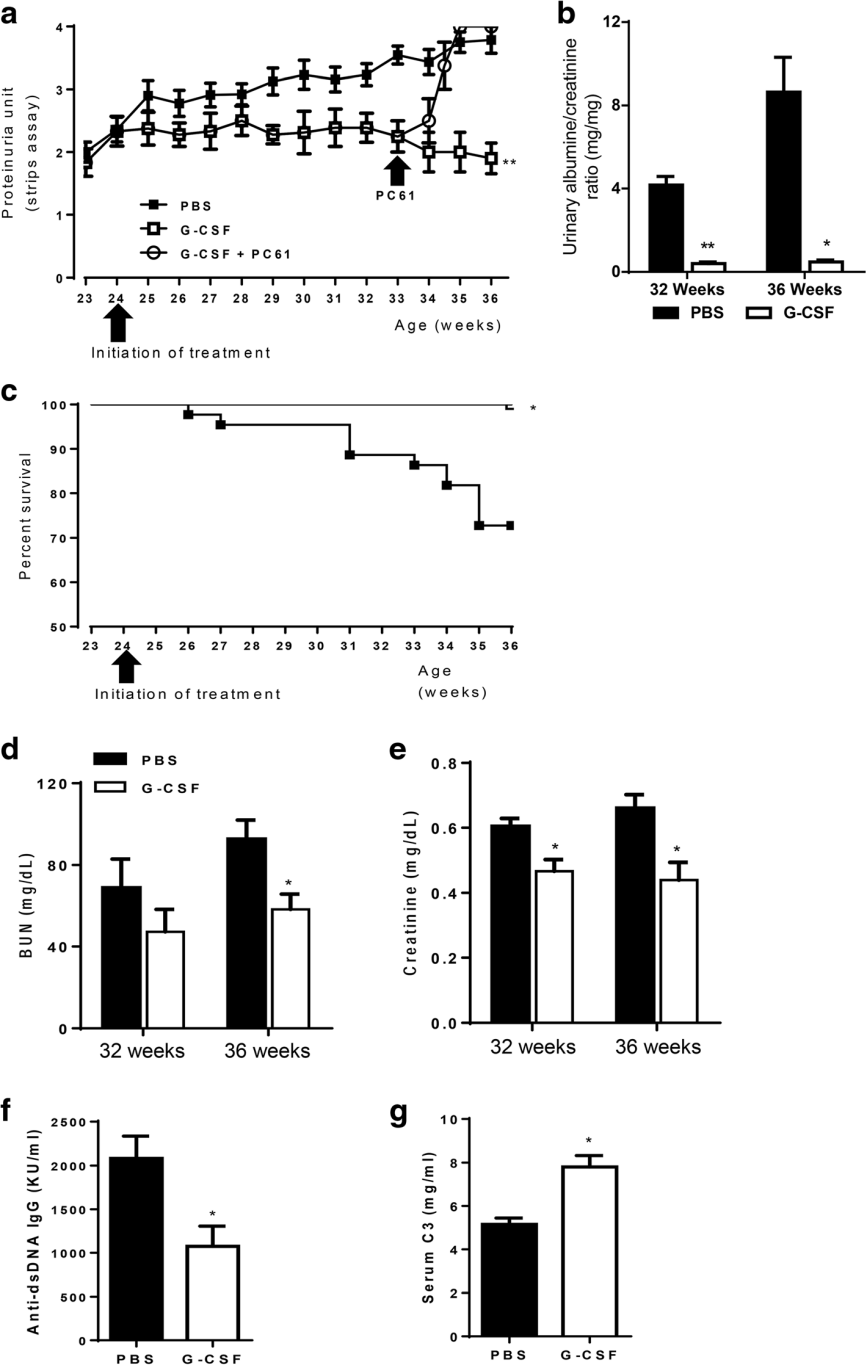
G-CSF treatment ameliorated disease activity, proteinuria, and mortality in lupus nephritis. **a** The G-CSF group had significantly lower proteinuria than the PBS group throughout the study period (*P* < 0.01, generalized estimating equation). When we decreased Tregs by PC61 administration at a dose of 0.5 mg 3 times a week during G-CSF treatment, Treg reduction abrogated beneficial effects of G-CSF on proteinuria. **b** Urine albumin/creatinine ratio was also significantly decreased at 32 (*P* < 0.01) and 36 weeks (*P* < 0.05) in the G-CSF group. **c** The G-CSF group had better survival rates than the PBS group (*P* < 0.05, log-rank test). **d**-**e** Serum levels of BUN (**d**) at 32 weeks and creatinine (**e**) at 32 and 36 weeks were significantly decreased by G-CSF treatment (*P* < 0.05, *t* test). **f** Serum levels of anti-dsDNA at 36 weeks were significantly lower in the G-CSF group (*P* < 0.05, *t*-test). **g** Serum C3 levels at 36 weeks were significantly higher in the G-CSF group (*P* < 0.05, *t*-test). The data are expressed as the mean ± standard error of the mean, with *n* = 14 − 16 mice per group. White and black bars indicate the G-CSF group and the PBS group, respectively. *P < 0.05, **P < 0.01 for comparisons between the G-CSF and the PBS groups. C3: complement component 3; G-CSF: granulocyte colony-stimulating factor; PBS: phosphate-buffered saline

Proteinuria from semi-quantitative measurement values was around 1.5 in most mice at 24 weeks of age. After 24 weeks, proteinuria gradually increased, reaching up to 3.3 in the PBS group. In contrast, proteinuria was maintained at a low level in the G-CSF group (*P* < 0.01, Fig. 1a). Urine albumin/creatinine ratio was significantly decreased at 32 weeks (*P* < 0.01) and 36 weeks (*P* < 0.05) in the G-CSF group (Fig. 1b). At 36 weeks of age, 27 % of mice in the PBS group had died, whereas there was no mortality in the G-CSF group (*P* < 0.05, Fig. 1c). Serum levels of BUN (Fig. 1d) and creatinine (Fig. 1e) were significantly lower in the G-CSF group compared with the PBS group (*P* < 0.05). G-CSF treatment decreased serum levels of anti-dsDNA IgG (*P* < 0.05, Fig. 1f) and increased serum levels of C3 (*P* < 0.05, Fig. 1g).

### G-CSF treatment attenuated renal pathologic injury

**Fig. 2:**
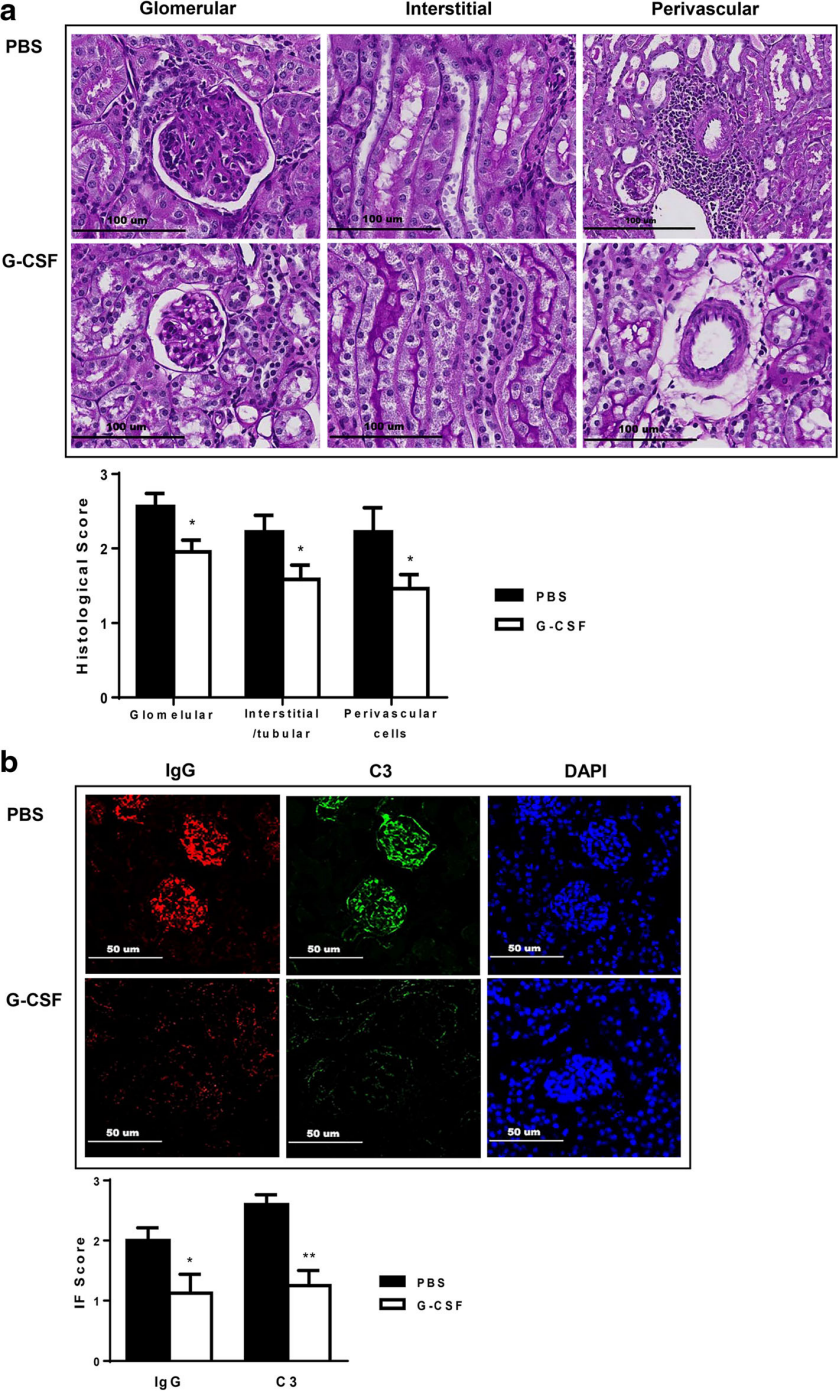
G-CSF treatment ameliorated histologic injury and immune deposits in lupus nephritis. **a** Representative kidney sections stained with PAS (200×; histological score on the right-panel). **b** Frozen sections stained for IgG and C3 deposits (200×; immunofluorescence staining score on the right-panel). Scores of both histologic injury and immune deposits were lower in the G-CSF group than the PBS group. Differences in histologic injury and immune deposit scores were analyzed by *t* tests. The data are expressed as the mean ± standard error of the mean, with *n* = 14 − 16 mice per group. White and black bars indicate the G-CSF group and the PBS group, respectively. *P < 0.05, **P < 0.01 for comparisons between the G-CSF and the PBS groups. C3: complement component 3; G-CSF, granulocyte colony-stimulating factor: PAS, periodic acid-Schiff’s reagent; PBS: phosphate-buffered saline

Renal injury in NZB/W F1 mice consisted of glomerular, interstitial, and perivascular infiltration of monocytes and lymphocytes, and concomitant immune complex deposition [21, 22]. NZB/W F1 mice in the PBS group exhibited severe renal injury, which was characterized by glomerulosclerosis, crescent formation, tubular cast deposition, increased mesangial matrix, and diffuse perivascular and interstitial mononuclear cell infiltration. All of these pathological injuries were attenuated in the G-CSF group (*P* < 0.05, Fig. 2a). G-CSF treatment also suppressed glomerular deposition of IgG (*P* < 0.05, Fig. 2b) and C3 (*P* < 0.01, Fig. 2b) in NZB/W F1 mice.

### G-CSF treatment suppressed renal infiltration of proinflammatory immune cells and decreased numbers of splenic immune cells

**Fig. 3:**
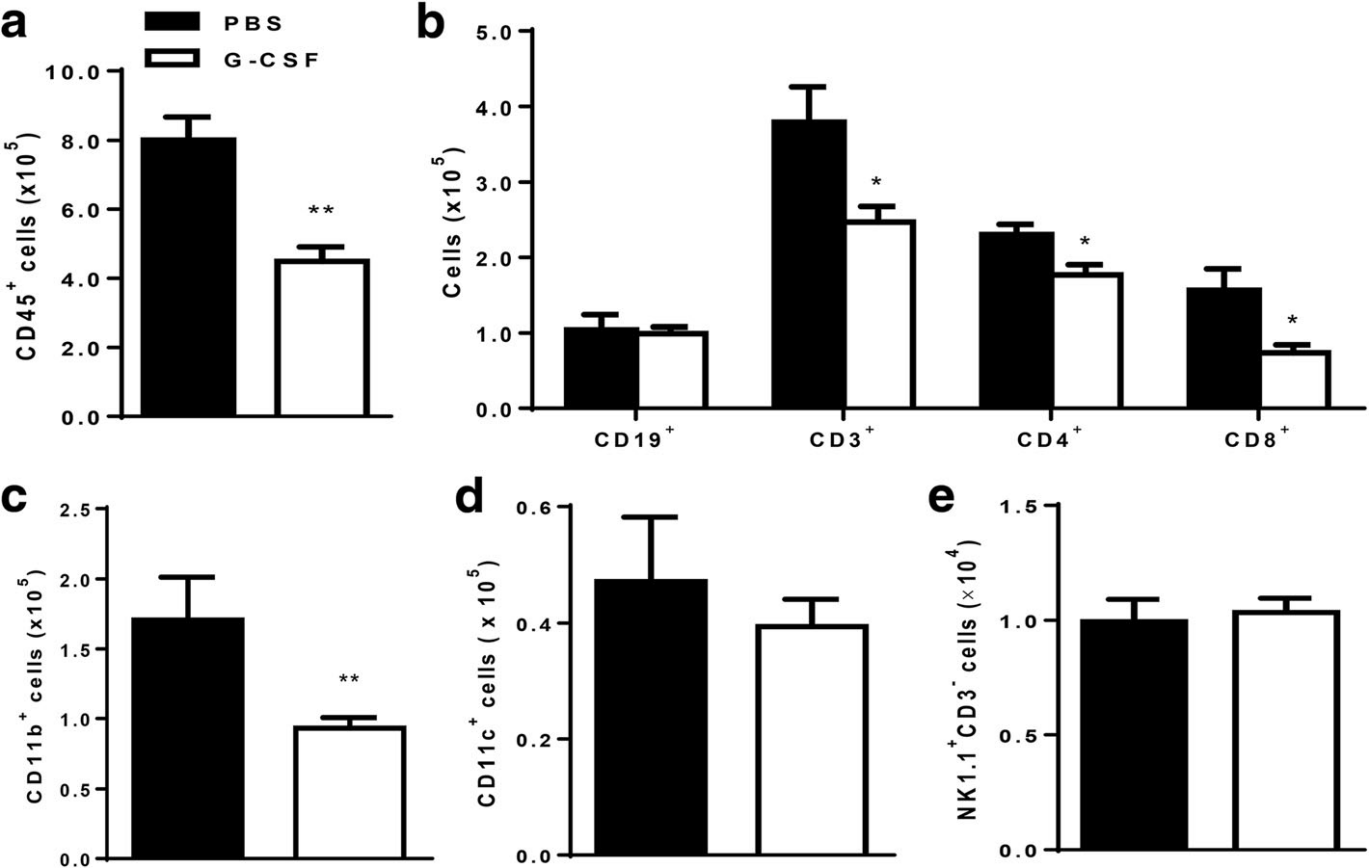
G-CSF treatment attenuated renal infiltration of inflammatory leukocytes. **a** Absolute number of total leukocytes (CD45^+^) in kidney was significantly decreased in the G-CSF group (*P* < 0.01). **b** Number of infiltrating T cells (CD3^+^) including both CD4^+^ and CD8^+^ T cells was decreased in the G-CSF group (*P* < 0.05), whereas number of infiltrating B cells (CD19^+^) was not decreased. **c** Numbers of CD11b^+^ myeloid cells were lower in the G-CSF group than in the PBS group (*P* < 0.01). **d**-**e** However, G-CSF did not decrease renal infiltration of either CD11c^+^ dendritic cells (**d**) or NK cells (NK1.1^+^CD3^-^, **e**). Flowcytometric analysis was used for quantifying renal cell types. The data are expressed as the mean ± standard error of the mean, with *n* = 4 − 5 mice per group. White and black bars indicate the G-CSF group and the PBS group, respectively. *P < 0.05, **P < 0.01 for comparisons between the G-CSF and the PBS groups using *t-*test. G-CSF: granulocyte colony-stimulating factor; PBS: phosphate-buffered saline

Renal infiltration of leukocyte subsets was analyzed in NZB/W F1 mice. We found that G-CSF treatment decreased renal infiltration of CD45^+^ leukocytes (*P* < 0.01, Fig. 3a), CD3^+^, CD4^+^ and CD8^+^ T cells (*P* < 0.05, Fig. 3b) when compared to that in the PBS group. Renal infiltration of myeloid cells (CD11b^+^) cells was also decreased in the G-CSF group (*P* < 0.01, Fig. 3c). However, numbers of renal CD19^+^ B cells (Fig. 3b), CD11c^+^ dendritic cells (Fig. 3d), and NK cells (NK1.1^+^CD3^-^, Fig. 3e) were similar between the two groups.

**Fig. 4:**
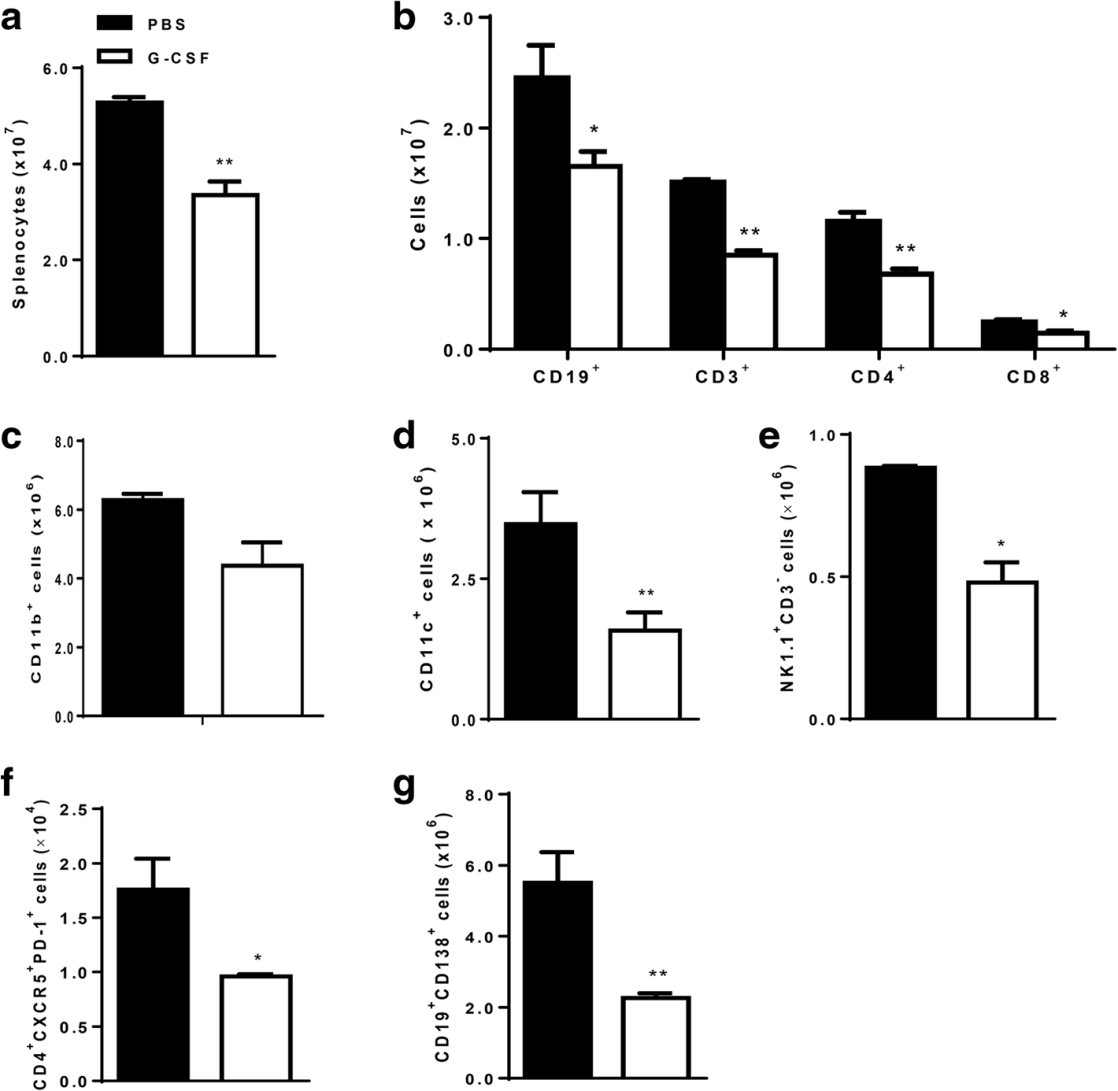
G-CSF treatment decreased numbers of splenic immune cells. **a** Absolute number of total splenocytes was lower in the G-CSF group (*P* < 0.01). **b** Numbers of B cell (CD19^+^), T cells (CD3^+^), and T cell subsets (CD4^+^_,_ CD8^+^) were decreased by G-CSF treatment. **c**-**e** Number of CD11b^+^ myeloid cells (**c**) was not significantly decreased by G-CSF, whereas numbers of CD11c^+^ dendritic cells (**d**) and NK cells (NK1.1^+^CD3^-^, **e**) were significantly lower in the G-CSF group. **f**-**g** Numbers of both follicular helper T cells (CD4^+^CXCR5^+^PD-1^+^, **f**) and plasma cells (CD19^low^CD138^hi^, **g**) were lower in the G-CSF group. Flowcytometric analysis was used for quantifying spleen cell types. The data are expressed as the mean ± standard error of the mean, with *n* = 4 − 5 mice per group. White and black bars indicate the G-CSF group and the PBS group, respectively. *P < 0.05, **P < 0.01 for comparisons between the G-CSF and the PBS groups using *t-*test. G-CSF: granulocyte colony-stimulating factor; PBS: phosphate-buffered saline

In the spleen, G-CSF treatment decreased the numbers of splenocytes (*P* < 0.01, Fig. 4a) including CD19^+^ B cells, CD3^+^ T cells, CD4^+^ T cells, and CD8^+^ T cells (Fig. 4b). Although there was no difference in the number of splenic CD11b^+^ myeloid cells (Fig. 4c), G-CSF treatment decreased numbers of splenic dendritic cells (*P* < 0.01, Fig. 4d) and NK cells (*P* < 0.05, Fig. 4e), when compared to the PBS group. In addition, the numbers of follicular helper T cells (CD4^+^CXCR5^+^PD-1^+^, *P* < 0.05, Fig. 4f) and plasma cells (CD138^hi^CD19^low^, *P* < 0.01, Fig. 4g) were also lower in the G-CSF group than in the PBS group.

### G-CSF treatment induced expansion of CD4^+^CD25^+^Foxp3^+^ Tregs

**Fig. 5:**
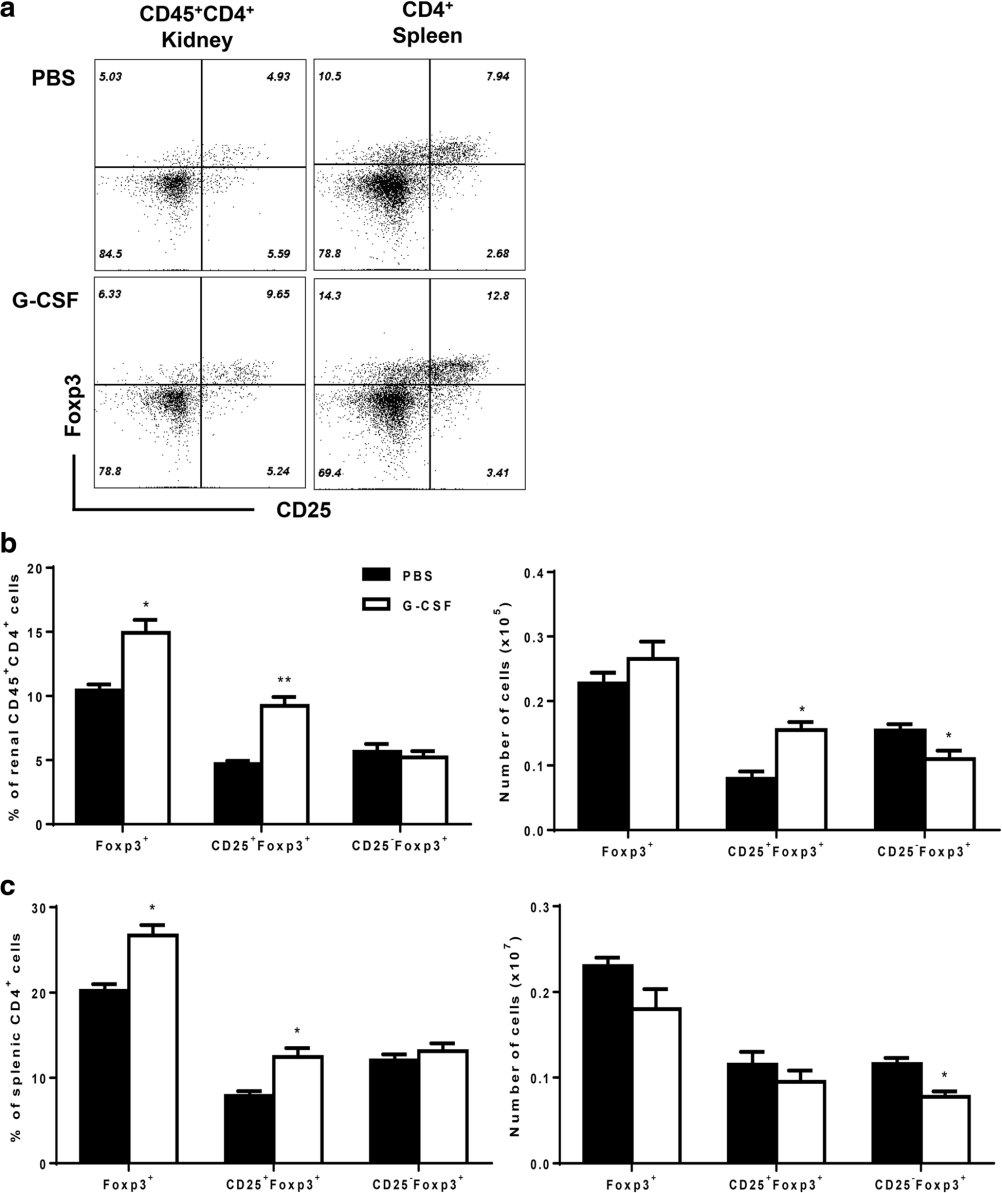
G-CSF treatment induced expansion of CD4^+^CD25^+^Foxp3^+^ regulatory T cells in both spleen and kidney. **a** Representative flow cytometric dot plots of CD25^+^Foxp3^+^ cells in kidney (CD45^+^CD4^+^ gated) and spleen (CD4^+^ gated). **b**-**c** Bar graphs showed proportions of Foxp3^+^, CD25^+^Foxp3^+^, and CD25^-^Foxp3^+^ among CD4^+^ T cells or absolute numbers of CD4^+^Foxp3^+^, CD4^+^CD25^+^Foxp3^+^, and CD4^+^CD25^-^Foxp3^+^ in kidneys (**b**) and spleen (**c**). The data are expressed as the mean ± standard error of the mean, with *n* = 4 − 5 mice per group. White and black bars indicate the G-CSF group and the PBS group, respectively. *P < 0.05, **P < 0.01 for comparisons between the G-CSF and the PBS groups using *t-*test. G-CSF: granulocyte colony-stimulating factor; PBS: phosphate-buffered saline; Tregs: regulatory T cells

The proportion and absolute numbers of CD4^+^CD25^+^Foxp3^+^ Tregs were evaluated in both the kidneys and spleen of NZB/W F1 mice (Fig. 5a-c). G-CSF treatment induced significant expansion of renal CD4^+^CD25^+^Foxp3^+^ Tregs in both proportion (*P* < 0.01, Fig. 5b) and absolute numbers (*P* < 0.05, Fig. 5b). Among CD4^+^Foxp3^+^ T cells in the kidney, the number of CD4^+^CD25^-^Foxp3^+^ cells was decreased by G-CSF treatment (*P* < 0.05, Fig. 5b). G-CSF treatment also increased the proportion of CD4^+^CD25^+^Foxp3^+^ Tregs in the spleen (*P* < 0.05, Fig. 5c). To assess whether Treg expansion is the main mechanism of G-CSF-mediated renal protection in NZB/W F1 mice, we decreased Tregs by PC61 treatment during G-CSF treatment (Additional file 1) and found that Treg reduction abrogated beneficial effects of G-CSF on proteinuria (Fig. 1a).

### G-CSF treatment decreased numbers of CD11b^+^Ly6G^+^Ly6C^low^ and CD11b^+^Ly6G^-^Ly6C^high^ myleoid cells

**Fig. 6:**
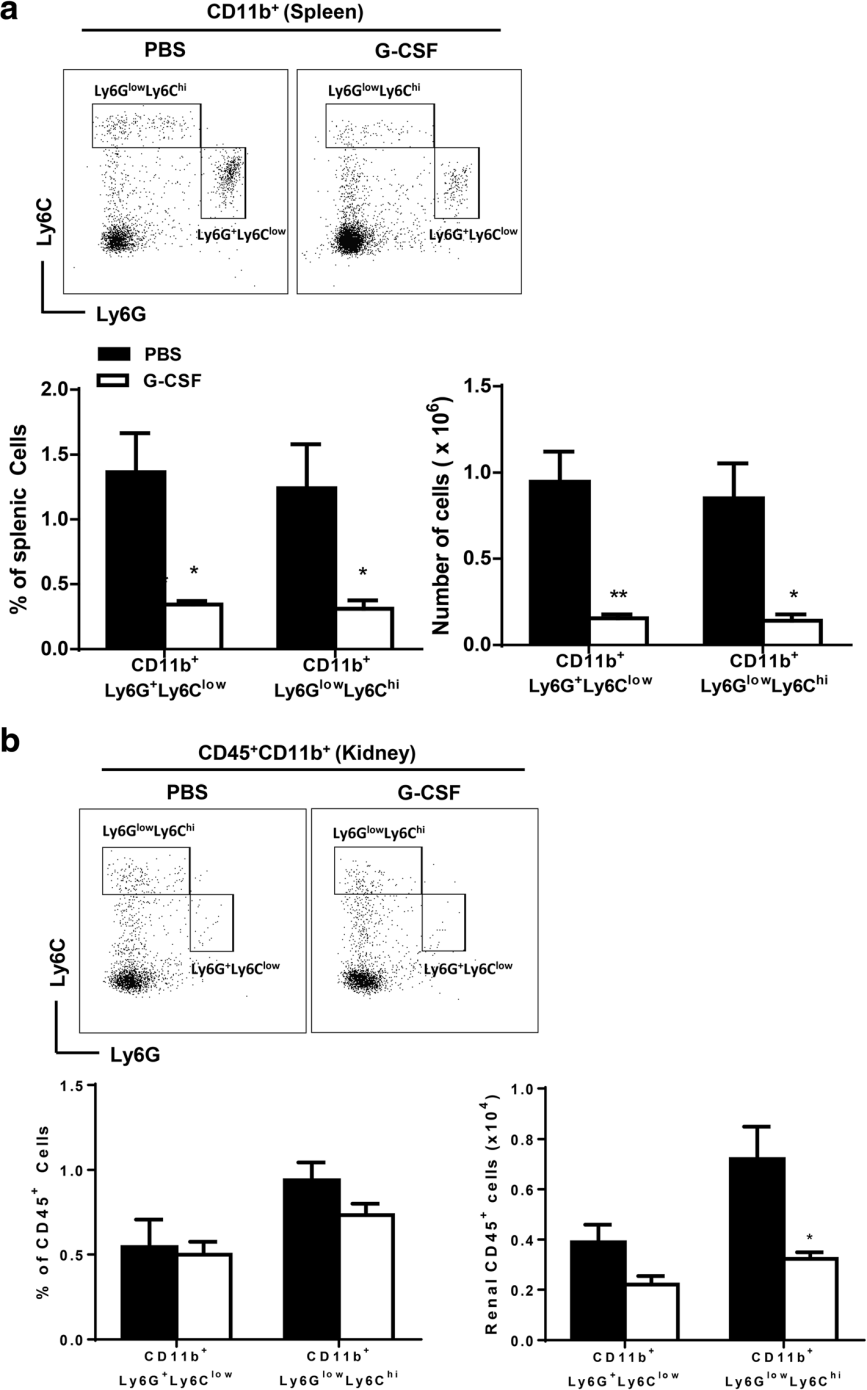
G-CSF treatment decreased numbers of CD11b^+^Ly6G^+^Ly6C^low^ and CD11b^+^Ly6G^-^Ly6C^high^ myeloid cells. **a** Flow cytometric dot plots for Ly6G and Ly6C defined CD11b^+^Ly6G^+^Ly6C^low^ and CD11b^+^Ly6G^-^Ly6C^high^ cells in spleen (CD11b^+^ gated). Bar graphs on the left side showed proportions of CD11b^+^Ly6G^+^Ly6C^low^ and CD11b^+^Ly6G^-^Ly6C^high^ cells among total splenocytes. Bar graphs on the right side indicated absolute numbers of CD11b^+^Ly6G^+^Ly6C^low^ and CD11b^+^Ly6G^-^Ly6C^high^ cells in spleen. **b** Flow cytometric dot plots for Ly6G and Ly6C defined CD11b^+^Ly6G^+^Ly6C^low^ and CD11b^+^Ly6G^-^Ly6C^high^ cells in kidney (CD45^+^CD11b^+^ gated). Bar graphs on the left side showed proportions of CD11b^+^Ly6G^+^Ly6C^low^ and CD11b^+^Ly6G^-^Ly6C^high^ cells among renal CD45^+^ cells. Bar graphs on the right side indicated absolute numbers of CD11b^+^Ly6G^+^Ly6C^low^ and CD11b^+^Ly6G^-^Ly6C^high^ cells in kidney. The data are expressed as the mean ± standard error of the mean, with *n* = 4 − 5 mice per group. White and black bars indicate the G-CSF group and the PBS group, respectively. *P < 0.05, **P < 0.01 for comparisons between the G-CSF and the PBS groups using *t-*test. G-CSF: granulocyte colony-stimulating factor; PBS: phosphate-buffered saline; Tregs: regulatory T cells

Among CD45^+^CD11b^+^ myeloid cells, Ly6G^-^Ly6C^high^ cells are considered to be inflammatory monocytes or monocytic MDSCs, and Ly6G^+^Ly6C^low^ cells are inflammatory granulocytes or granulocytic MDSCs. In spleen, G-CSF treatment decreased proportions and absolute numbers of both Ly6G^+^Ly6C^low^ and Ly6G^-^Ly6C^high^ cells (Fig. 6a). Furthermore, G-CSF treatment decreased renal infiltration of Ly6G^+^Ly6C^low^ and Ly6G^-^Ly6C^high^ cells; however, impact on Ly6G^+^Ly6C^low^ was not statistically significant (Fig. 6b).

### G-CSF treatment suppressed expression of proinflammatory cytokines in NZB/W F1 mice

**Fig. 7:**
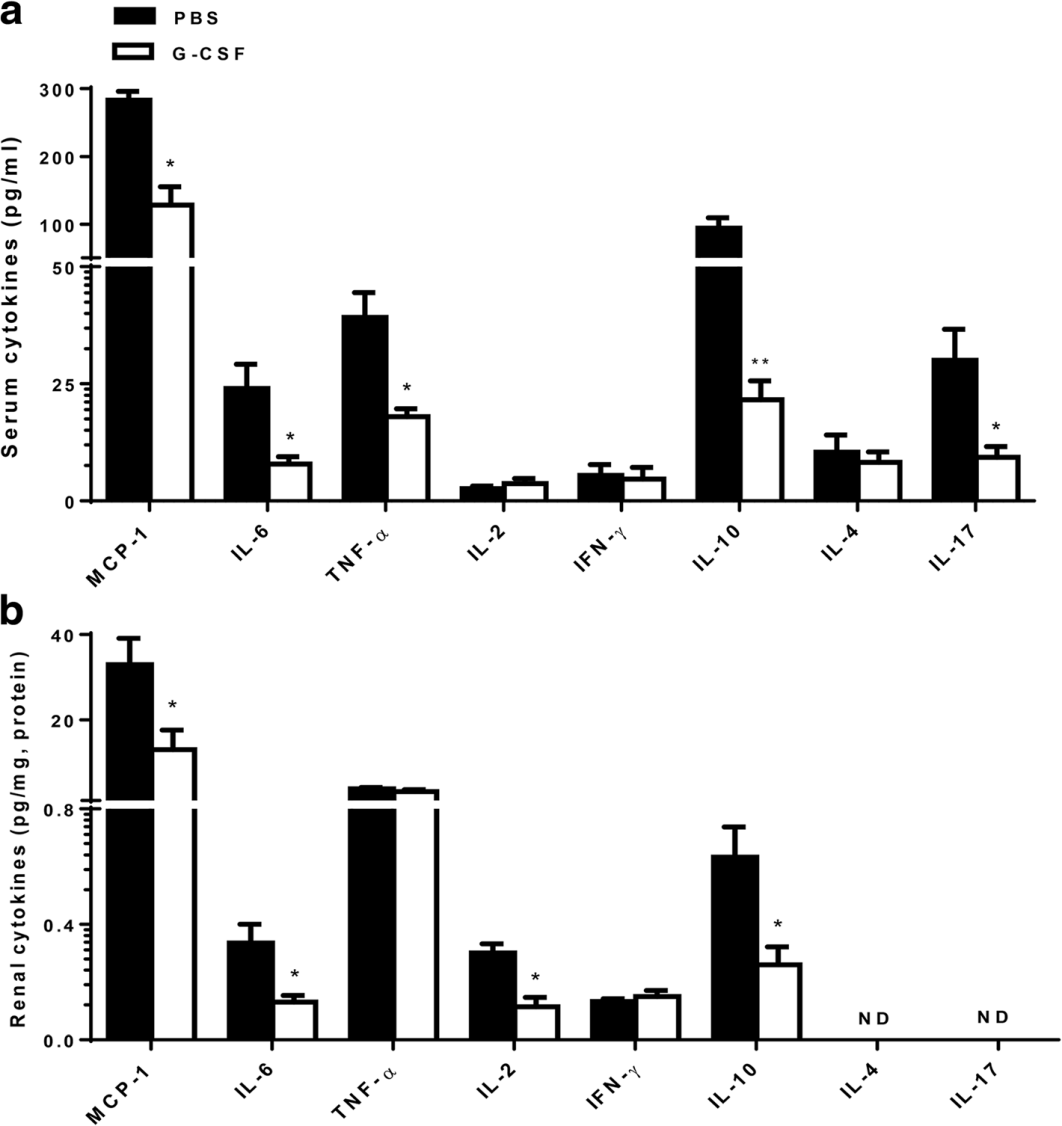
G-CSF treatment decreased levels of proinflammatory chemokine and cytokines in the serum and kidneys. Expression levels of proinflammatory chemokine (MCP-1), proinflammatory cytokines (IL-6, TNF-α), Th1 cytokines (IL-2, INF-γ), Th2 cytokines (IL-4, IL-10), and Th17 cytokines (IL-17A) were measured in serum (**a**) and renal tissues (**b**). G-CSF treatment significantly decreased serum levels of MCP-1, IL-6, TNF-α, IL-10 and IL-17. In renal tissues, G-CSF treatment significantly decreased levels of MCP-1, IL-6, IL-2, and IL-10. The data are expressed as the mean ± standard error of the mean, with *n* = 4 − 7 mice per group. White and black bars indicate the G-CSF group and the PBS group, respectively. *P < 0.05, **P < 0.01 for comparisons between the G-CSF and the PBS groups using *t-*test. G-CSF: granulocyte colony-stimulating factor; IFN: interferon; IL: interleukin; MCP-1: monocyte chemoattractant protein-1; PBS: phosphate-buffered saline; Th: helper T cells; TNF: tumor necrosis factor; Tregs: regulatory T cells

G-CSF treatment decreased levels of MCP-1, IL-6, and TNF-α in either serum (Fig. 7a) or renal tissues (Fig. 7b). G-CSF treatment also decreased expression of IL-2, IL-10, and IL-17, which are important type 1 helper T cells (Th1), type 2 helper T cells (Th2), and type 17 helper T cells (Th17) cytokines, respectively. Overall, G-CSF treatment decreased expression of proinflammatory chemokine and cytokines in renal tissues as well as systemically in NZB/W F1 mice.

### Low-dose G-CSF treatment did not expand regulatory T cells or ameliorate lupus nephritis

When we administered 250 μg/kg of G-CSF 3 times a week, G-CSF did not attenuate proteinuria (Additional file 2a) or mortality (Additional file 2b). There was no significant difference in either anti-ds DNA levels (Additional file 2c) or serum C3 levels (Additional file 2d). Histologic injury was also not improved (Additional file 2e). In parallel, Tregs were not expanded in kidneys (Additional file 2f) or spleen (Additional file 2g). Low-dose GCF treatment did not decrease inflammatory granulocytes (Additional file 2h-i).”

## Discussion

This study demonstrated that G-CSF treatment decreased proteinuria, serum levels of BUN and creatinine, and ultimately decreased mortality of NZB/W F1 mice through the expansion of Tregs. G-CSF treatment also decreased serum levels of anti-dsDNA, increased serum levels of C3, and attenuated renal tissue injury including deposition of IgG and C3 in NZB/W F1 mice. Furthermore, G-CSF treatment induced expansion of CD4^+^CD25^+^Foxp3^+^ Tregs, with decreased renal infiltration of T cells, B cells, inflammatory granulocytes and monocytes rather than MDSCs. G-CSF treatment also decreased expression of MCP-1, IL-6, TNF-α, IL-2, IL-10, and IL-17.

Chronic treatment of low-dose G-CSF (10 μg/kg) aggravated lupus nephritis in MRL/*lpr* mice, where these results might be attributed to increased Th2 cytokines and IFN-α [3, 7, 13]. Conversely, chronic treatment of high-dose G-CSF (200 μg/kg) attenuated lupus nephritis in MRL/*lpr* mice by potentially decreasing glomerular expression of FcRγIII and IL-12 production [13]. The G-CSF dose in this study was comparable to the high-dose of G-CSF in the previous study [13]. When we reduced G-CSF dose by 40 %, we could not ameliorate lupus nephritis. Proinflammatory chemokines (MCP-1) and cytokines (IL-6, TNF-α), Th1 cytokines (IL-2, IFN-γ), Th2 cytokines (IL-4, IL-10), and Th17 cytokines (IL-17A) are involved in pathogenesis of lupus nephritis, and are therapeutic targets for lupus nephritis [23, 24]. G-CSF treatment decreased the levels of these pathogenic chemokine/cytokines such as MCP-1, IL-6, TNF-α, IL-2, IL-10, and IL-17, and thus were consistent with its suppressive effects on both innate and adaptive immune cells in NZB/W F1 mice. Despite reduced IL-10 levels, reduced levels of IL-6 in both serum and renal tissues might have contributed towards Treg expansion by G-CSF treatment. Taken together, these results indicated that the suppression of various proinflammatory cytokines involved in lupus nephritis pathogenesis could have led to the G-CSF-mediated attenuation of lupus nephritis.

G-CSF decreased inflammatory granulocytes (CD11b^+^Ly6G^+^Ly6C^low^) or monocytes (CD11b^+^Ly6G^-^Ly6C^high^) in both kidney and spleen. These results were consistent with previous studies in murine lupus models. In those studies, the percentage of CD11b^+^Gr-1^low^ monocytes in the kidney increased as the disease progressed in the MRL/lpr mouse model of lupus, and it expanded in B6 mice with established chronic graft-versus-host disease [12, 25]. Although inflammatory granulocytes and monocytes share surface markers with the MDSCs, these data suggested that G-CSF treatment decreased inflammatory granulocytes and monocytes rather than MDSCs in parallel with improvement of lupus nephritis.

We found that Tregs expanded in the G-CSF group and Treg depletion abrogated the G-CSF-mediated beneficial effects on proteinuria in NZB/W F1 mice. These findings were consistent with a previous study, which demonstrated that G-CSF recruited Tregs, and thereby prevented onset of spontaneous diabetes in NOD mice [26]. CD4^+^CD25^+^ Treg cells were decreased in SLE patients and Treg cell numbers were inversely correlated with disease activity [27, 28]. Moreover, infusion of Tregs attenuated disease progression and reduced mortality in murine lupus [29]. Therefore, we assumed that Treg expansion could be the beneficial mechanism of G-CSF on lupus nephritis in this study. Although MDSC could contribute to induction of Tregs [30], lack of MSDC expansion might exclude this mechanism in our setting. It was also observed that G-CSF decreased CD25^-^Foxp3^+^ T cells in contrast to CD25^+^Foxp3^+^ Tregs. These results demonstrated another benefit of G-CSF treatment for lupus nephritis, because CD25^-^Foxp3^+^ T cells were increased in the case of active lupus nephritis and had lower suppressive activity than CD25^+^Foxp3^+^ Tregs [31, 32].

Beneficial effects of G-CSF for lupus nephritis observed in this study suggest G-CSF as potential treatment for lupus nephritis, considering G-CSF is already used in clinical treatment of neutropenia after chemotherapy. Human G-CSF is less potent in mice than murine G-CSF, thus we used a very high dose of human G-CSF in NZB/W F1 mice. We expect to reduce this dose to tolerable levels in clinical trials.

Most patients with lupus nephritis take long-term immunosuppressants such as corticosteroids, cyclophosphamide, or mycophenolate mofetil. Therefore, one of the limitations of this study is that we did not evaluate the interacting effects of G-CSF with standard immuosuppressants for lupus nephritis. Further studies are needed to confirm our findings in clinically plausible situations.

## Conclusions

G-CSF treatment ameliorated lupus nephritis, and preferential expansion of CD4^+^CD25^+^Foxp3^+^ Tregs instead of MDSC may be the underlying mechanism. Therefore, G-CSF may have a therapeutic potential for lupus nephritis.

## Additional files

Additional file 1: PC61 treatment markedly decreased regulatory T cells in both kidneys and spleen. During G-CSF treatment, we administered anti-CD25 depleting antibodies (PC61) at a dose of 0.5 mg 3 times a week from 33 weeks to 36 weeks. At harvest, regulatory T cells were depleted in spleen and markedly decreased in kidneys in the G-CSF plus PC61 group. (TIF 4173 kb)
Additional file 2: Low-dose G-CSF treatment did not expand regulatory T cells or ameliorate lupus nephritis. When we administered 250 μg/kg of G-CSF 3 times a week, G-CSF did not attenuate proteinuria (a) or mortality (b). There was no significant difference in either anti-ds DNA levels (c) or serum C3 levels (d). Histologic injury was also not improved (e). In parallel, Tregs were not expanded in kidneys (f) or spleen (g). Low-dose GCF treatment did not decrease inflammatory granulocytes or monocytes in kidney (h) or spleen (i). (TIF 2014 kb)

## Abbreviations

Anti-dsDNA: Anti-double stranded DNA
C3: Complement 3
G-CSF: Granulocyte colony-stimulating factor
IFN: Interferon
IL: Interleukin
MDSC: Myeloid-derived suppressor cells
PAS: Periodic acid-Schiff’s
PBS: Phosphate-buffered saline
SLE: Systemic lupus erythematosus
TNF: Tumor necrosis factor
Tregs: Regulatory T cells
