# Supplementary figures and images for: Granulocyte colony-stimulating factor treatment ameliorates lupus nephritis through the expansion of regulatory T cells
Source: BMC Nephrol. 2016 Nov 15;17:175. doi: 10.1186/s12882-016-0380-x (PMC5111287; doi:10.1186/s12882-016-0380-x)

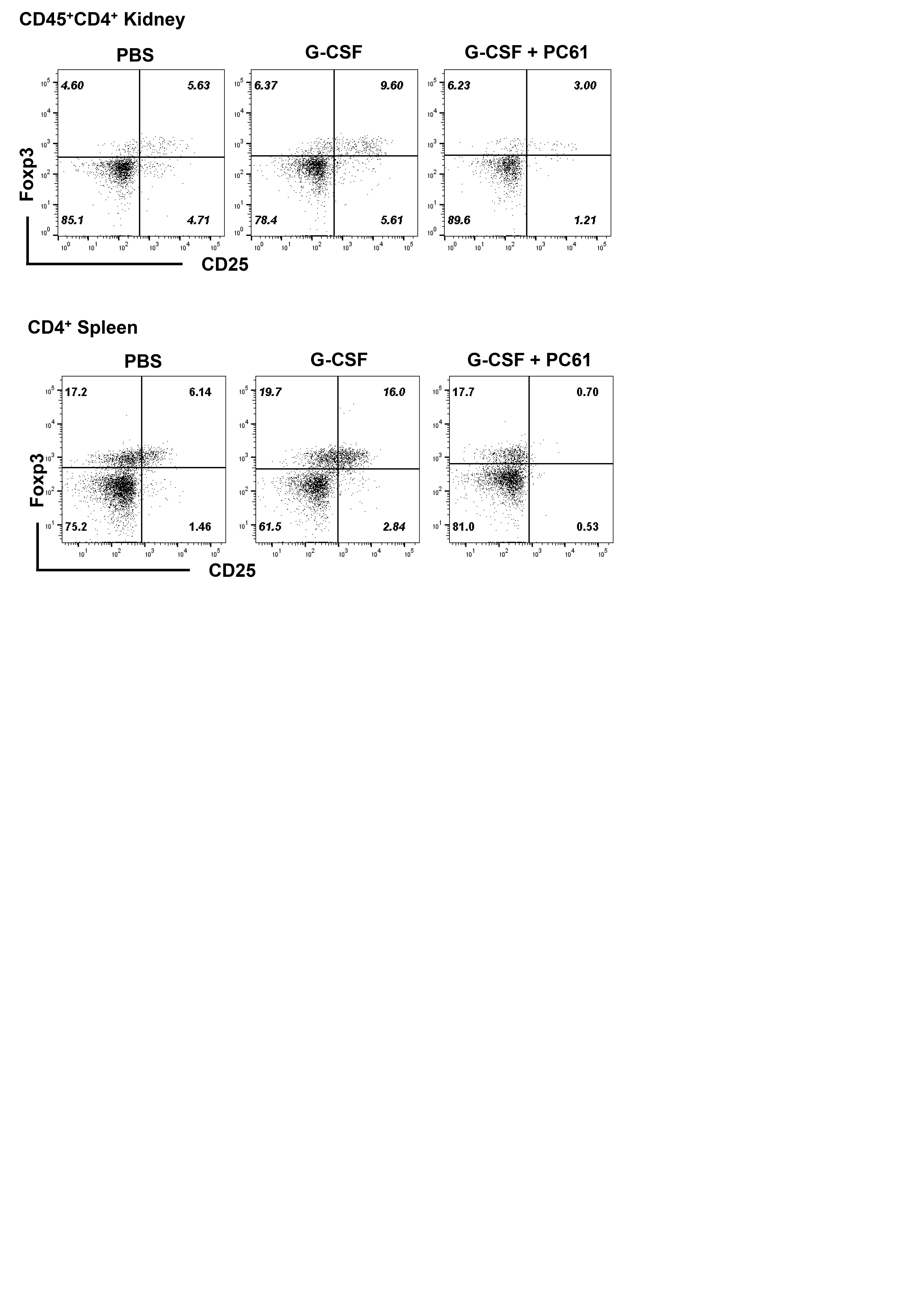

Supplement: Additional file 1: — PC61 treatment markedly decreased regulatory T cells in both kidneys and spleen. During G-CSF treatment, we administered anti-CD25 depleting antibodies (PC61) at a dose of 0.5 mg 3 times a week from 33 weeks to 36 weeks. At harvest, regulatory T cells were depleted in spleen and markedly decreased in kidneys in the G-CSF plus PC61 group. (TIF 4173 kb) [file 12882_2016_380_MOESM1_ESM.tif]

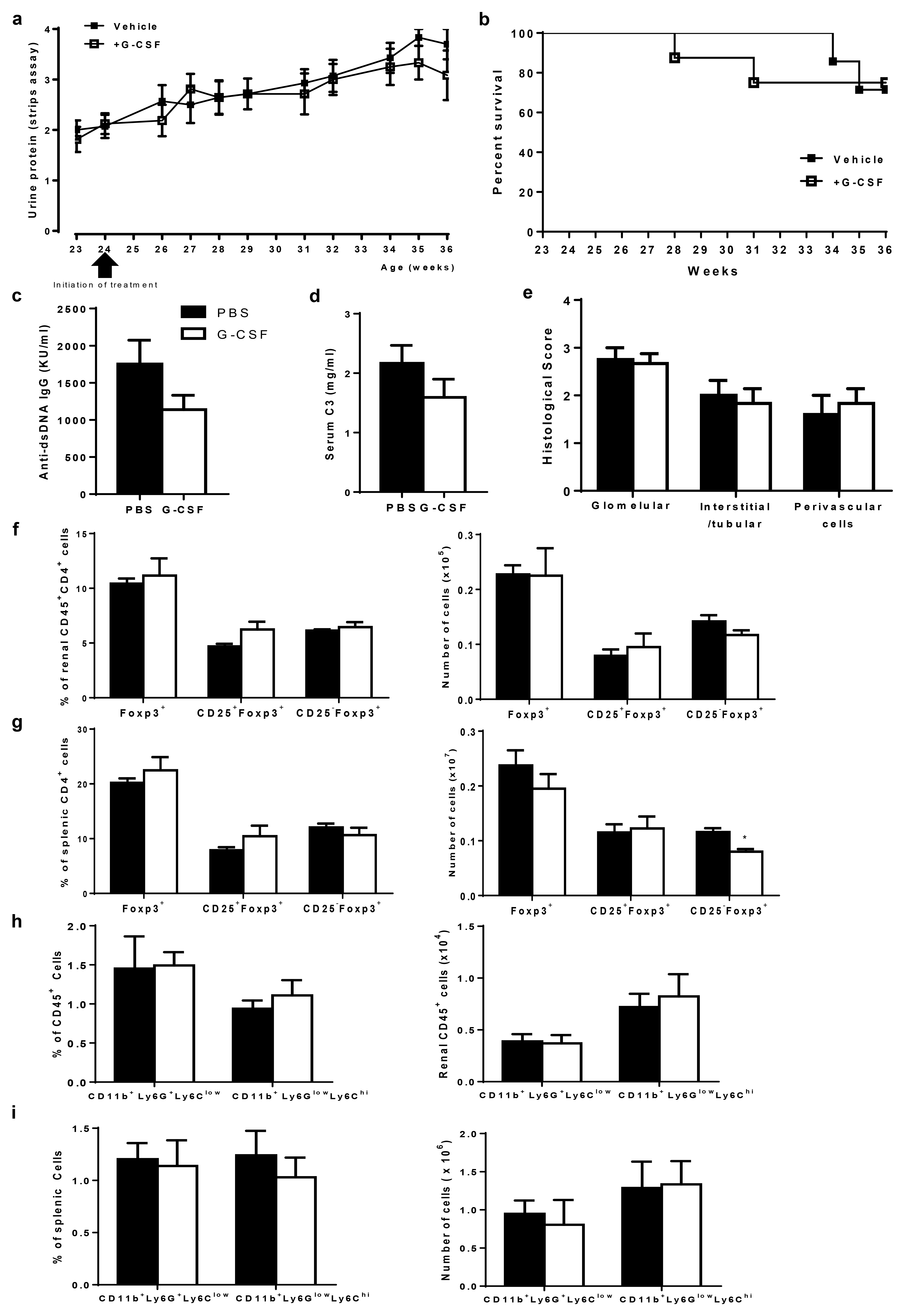

Supplement: Additional file 2: — Low-dose G-CSF treatment did not expand regulatory T cells or ameliorate lupus nephritis. When we administered 250 μg/kg of G-CSF 3 times a week, G-CSF did not attenuate proteinuria (a) or mortality (b). There was no significant difference in either anti-ds DNA levels (c) or serum C3 levels (d). Histologic injury was also not improved (e). In parallel, Tregs were not expanded in kidneys (f) or spleen (g). Low-dose GCF treatment did not decrease inflammatory granulocytes or monocytes in kidney (h) or spleen (i). (TIF 2014 kb) [file 12882_2016_380_MOESM2_ESM.tif]
